# Evaluating the safety and feasibility of remote ischemic conditioning for slowing cognitive decline in mild Alzheimer’s dementia

**DOI:** 10.3389/fneur.2025.1592829

**Published:** 2025-06-24

**Authors:** Xin Huang, Qiling Ji, Tanna Tong, Lipeng Cai, Omar Elmadhoun, Yanfang Zeng, Xiaokun Geng, Yuchuan Ding

**Affiliations:** ^1^Department of Neurology, Beijing Luhe Hospital, Capital Medical University, Beijing, China; ^2^Luhe Institute of Neuroscience, Capital Medical University, Beijing, China; ^3^Division of Critical Care Medicine, Mayo Clinic, Rochester, MN, United States; ^4^Department of Neurology, Wayne State University School of Medicine, Detroit, MI, United States

**Keywords:** Alzheimer’s disease, non-pharmacological intervention, cognition disorders, neurodegenerative biomarkers, multi-target intervention

## Abstract

**Objective:**

Alzheimer’s disease (AD) is characterized by complex pathological mechanisms involving neuroinflammation, oxidative stress, and vascular dysfunction. Remote Ischemic Conditioning (RIC) has shown potential in addressing these pathways by improving cerebral blood flow, reducing oxidative stress, and modulating inflammatory responses. This protocol focuses on evaluating the safety, feasibility, and preliminary efficacy of RIC as a multi-target intervention for delaying cognitive decline in patients with mild Alzheimer’s dementia, aiming to improve cognitive outcomes and overall quality of life.

**Methods and expected results:**

This study is a randomized, controlled, single-center, prospective clinical trial designed to evaluate the safety, feasibility, and preliminary efficacy of RIC in patients with mild Alzheimer’s dementia. Eligible participants will be recruited and randomly assigned to either the RIC group or a control group receiving sham RIC, with 20 patients in each group. Participants will receive either RIC or sham RIC once daily over a 3-month period. Outcome measures will assess cognitive function, psychological well-being, and inflammatory and neurodegenerative biomarkers. Psychiatric adverse events will be monitored throughout the treatment using the Hamilton Anxiety Rating Scale (HAMA) and the Hamilton Depression Rating Scale (HAMD-17). Cognitive function and daily living abilities will be evaluated at baseline, 3 months, 6 months, and 12 months post-treatment using the Mini-Mental State Examination (MMSE), Montreal Cognitive Assessment (MoCA), Clinical Dementia Rating (CDR), and the Activities of Daily Living (ADL) scales. In addition, blood samples will be collected at each time point to measure plasma biomarkers of *β*-amyloid species and serum inflammatory cytokines to assess potential changes in cognitive decline, disease progression, and inflammation. The primary endpoint is safety, with the expectation that RIC will not increase psychiatric adverse events as reflected in HAMA and HAMD-17 scores. Primary efficacy endpoints include improvements in MMSE, MoCA, CDR, and ADL scores, indicating potential cognitive benefits and enhanced daily functioning. Secondary endpoints will analyze biomarkers to evaluate disease progression and inflammation levels before and after treatment.

**Conclusion:**

This trial aims to determine the safety, feasibility, and potential effectiveness of RIC as a multi-target intervention for mild Alzheimer’s dementia by integrating cognitive and neuropsychological assessments with biological markers, providing a foundation for future studies.

## Introduction

With the global population aging, Alzheimer’s disease (AD) has become one of the most prevalent neurodegenerative disorders, posing a substantial challenge to healthcare systems. The prevalence of AD is approximately 5% among individuals aged 65 and older, rising to 33.3% in those aged 85 and above (1). As this demographic shift continues, the development of effective prevention and treatment strategies for AD grows increasingly urgent (2, 3).

The hallmark pathological features of AD include the deposition of *β*-amyloid (Aβ) plaques and tau protein tangles in the brain (4). Its progression involves multiple interconnected mechanisms, including neuroinflammation, oxidative stress, and vascular abnormalities, which contribute to the disease’s complexity (5–7). Most approved treatments for AD focus on symptom management rather than altering disease progression (8, 9). Newer approaches, such as anti-amyloid monoclonal antibodies, have shown potential in modifying disease progression but are limited by side effects and challenges in crossing the blood–brain barrier (2, 8–11). Given the multifaceted nature of AD, multi-target interventions that address multiple pathways may be more effective in slowing disease progression (12).

Early diagnosis and intervention are essential, especially in mild Alzheimer’s dementia, where symptoms remain subtle but represent a critical window for therapeutic intervention (13). Treating AD in its mild stage may help prevent the disease’s transition to more severe stages, ultimately preserving cognitive function and reducing the burden on healthcare resources (4, 11, 14–16).

Remote ischemic conditioning (RIC) is a non-pharmacological, non-invasive intervention that has shown promise in activating neuroprotective signaling pathways (15, 17, 18). By enhancing cerebral blood flow, reducing oxidative stress, and modulating inflammation, RIC has demonstrated potential in preventing cognitive decline and improving cognitive function in conditions like vascular cognitive impairment (VCI) (15, 16, 19, 20). Given its multi-target mechanism, RIC could also offer neuroprotective benefits in AD by addressing similar pathophysiological processes (12, 20–22).

This early-phase trial is designed to assess the safety, feasibility, and preliminary efficacy of RIC in slowing cognitive decline in patients with mild Alzheimer’s dementia. By evaluating the tolerability and safety of daily RIC over 3 months and its effects on plasma biomarkers and inflammatory factors, this study aims to lay the groundwork for future research on RIC as a potential intervention for AD-related cognitive decline.

## Subjects and methods

### Study design

This study was registered on www.chictr.org.cn in November 2024 (ID: ChiCTR2400092245) and approved by the Ethics Committee of Beijing Luhe Hospital, Capital Medical University, China (2024-LHKY-092-03). All study participants were fully informed about the research process, risks, and benefits, and voluntarily agreed to participate by signing an informed consent form. The study protocol and informed consent form were approved by the Ethics Committee.

A total of 40 participants will be randomly assigned to either the observation group (RIC group) or the control group (sham RIC group), with 20 participants in each group. All participants with AD included in the study will continue receiving standard pharmacotherapy in accordance with established clinical practice guidelines. Each group will begin RIC or sham treatment, administered once daily for 45 min per session over a period of 3 months.

Specifically, our study has established a Data and Safety Monitoring Board (DSMB) composed of independent experts to ensure rigorous oversight of participant safety, data integrity, and scientific validity throughout the trial. The DSMB is responsible for regularly reviewing accumulated safety data, monitoring adverse events, and making recommendations regarding study continuation or modification as needed to protect participant welfare.

### Patient population

Participants will be recruited from the Neurology Outpatient Clinic and Inpatient Department of Beijing Luhe Hospital, Capital Medical University. Inclusion criteria include: (1) a diagnosis of mild Alzheimer’s dementia based on the 2024 core clinical criteria for “Stage 4 Dementia with mild functional impairment” by the National Institute on Aging and Alzheimer’s Association (NIA-AA) (8); (2) objective evidence of cognitive impairment; and (3) cranial CT or MRI scans supporting AD diagnosis.

Exclusion criteria include: (1) the presence of other conditions that could explain memory impairment such as severe anxiety and depression; (2) contraindications to ischemic conditioning, including severe soft tissue injury, fractures, or bilateral peripheral vascular disease of the upper limbs; (3) hemodynamic instability, defined as systolic blood pressure >180 mmHg, diastolic blood pressure >110 mmHg, heart rate <40 bpm or >100 bpm, peripheral oxygen saturation ≤92%, or body temperature ≥38.5°C; (4) life expectancy ≤1 year; (5) poor tolerance to treatment due to circulatory, respiratory, digestive, hematological diseases, or malignancies; (6) coagulation disorders or active bleeding; (7) concurrent inflammatory conditions or use of medications affecting inflammatory cytokine levels; (8) cerebrovascular diseases, including cerebral infarction, cerebral hemorrhage, vascular dementia and small vessel disease, as well as other neurological or systemic conditions such as thyroid dysfunction, epilepsy, Parkinson’s disease, malnutrition, chronic diarrhea, carbon monoxide poisoning, traumatic brain injury, alcohol intoxication, or other serious physical illnesses; and (9) participation in another ongoing clinical trial.

### Randomization

This study employs a single-center, randomized controlled, prospective design to ensure scientific rigor and precision. Eligible patients will be randomly assigned to either the RIC group or the sham RIC group. Randomization will be conducted using opaque envelopes. Sealed, numbered, opaque envelopes containing the group assignments will be opened by a researcher not involved in the study design or data analysis, in the order of participant enrollment. The subjects, researchers, and evaluators will be blinded to the treatment allocation.

### Interventions

**Figure 1:**
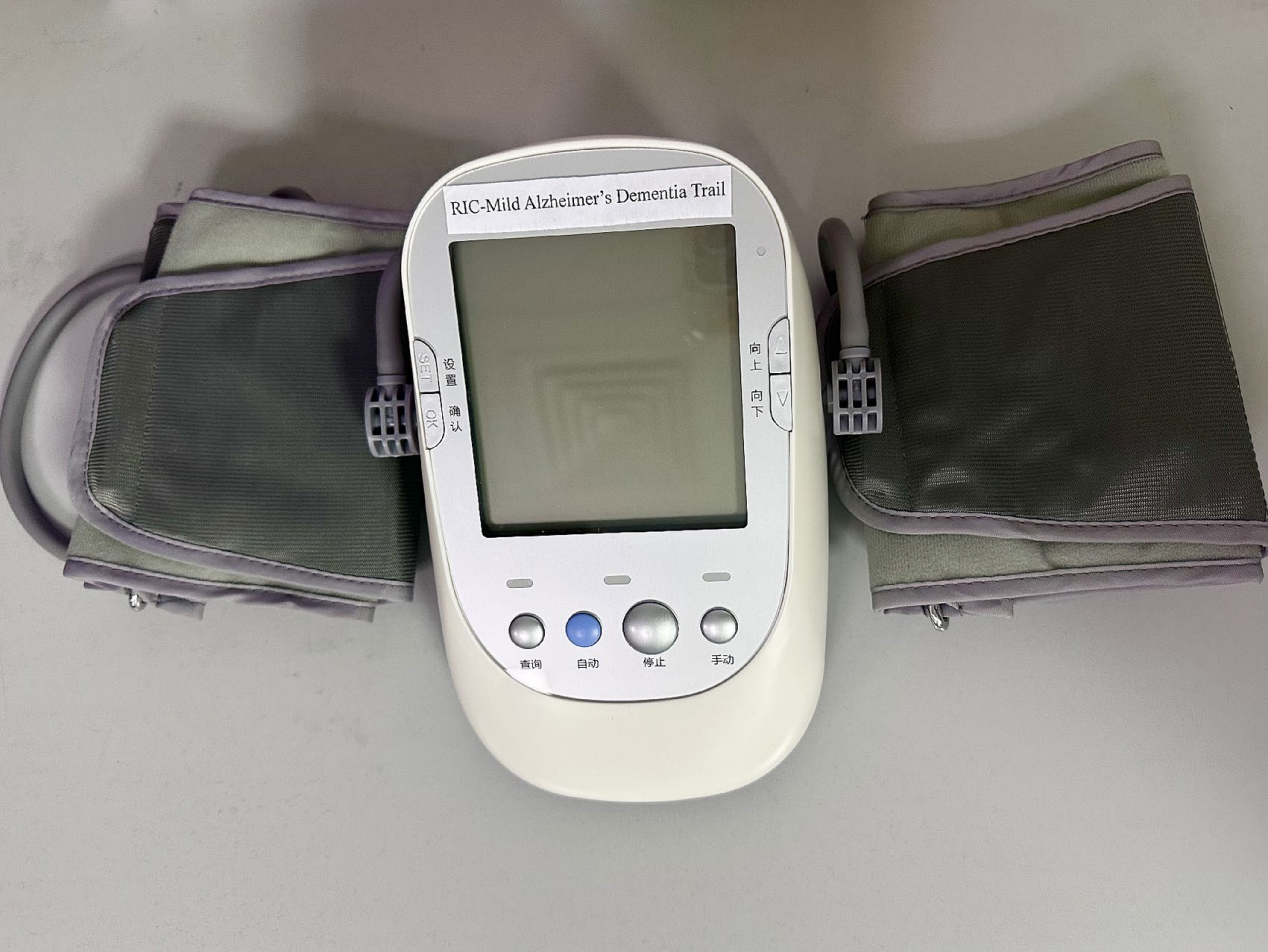
Device for remote ischemic conditioning.

All study participants will undergo bilateral limb RIC using the Doctormate IPC-906, manufactured by Beijing Renqiao Neuroscience Institute. Electronic tourniquet cuffs will be placed on both arms of each participant (Figure 1).

Lacking clinical RIC studies in AD populations, there are no intervention data available for direct reference. In a study published in Lancet Neurology, Dr. Jixunming and colleagues demonstrated that a 45 min daily RIC protocol significantly reduced the recurrence of strokes and the incidence of cardiovascular events in individuals with intracranial atherosclerotic stenosis (23). Additionally, AD research on Transcranial Magnetic Stimulation and Transcranial Direct Current Stimulation has shown cognitive improvements with 2 to 6 months of non-pharmacological treatment (24, 25). As an initial to evaluate safety, the duration of RIC in this study has been set at 3 months. In the RIC group, each session will involve five cycles of cuff inflation to 200 mmHg for 5 min, followed by a 5 min deflation period, conducted daily for 45 min. The sham RIC group will follow an identical schedule, with cuff inflation limited to 60 mmHg for 5 min, followed by a 5 min deflation period, repeated five times per session, once daily, for 45 min. The inflation pressure of 60 mmHg was specifically chosen for the sham group based on previous experience and published protocols, as this level of cuff inflation generates sufficient pressure to mimic the sensation of treatment without inducing true limb ischemia or producing significant physiological changes. Participants undergoing sham procedures typically report a credible sensation similar to active treatment, supporting its validity as a placebo. Additionally, this pressure level aligns with previously validated sham protocols used in multiple high-quality clinical trials (e.g., NCT03868007; ChiCTR2000041042), which have demonstrated strong blinding effectiveness and placebo credibility (26, 27).

The RIC intervention requires participants to attend their assigned community health service stations daily during designated time slots for RIC training. At these facilities, they will use specialized RIC equipment to conduct the sessions as outlined in the study protocol. A dedicated team of specialists will be available to provide comprehensive guidance and support throughout the process, ensuring participant safety, promoting treatment adherence, and maintaining the efficacy of the sessions.

**Figure 2:**
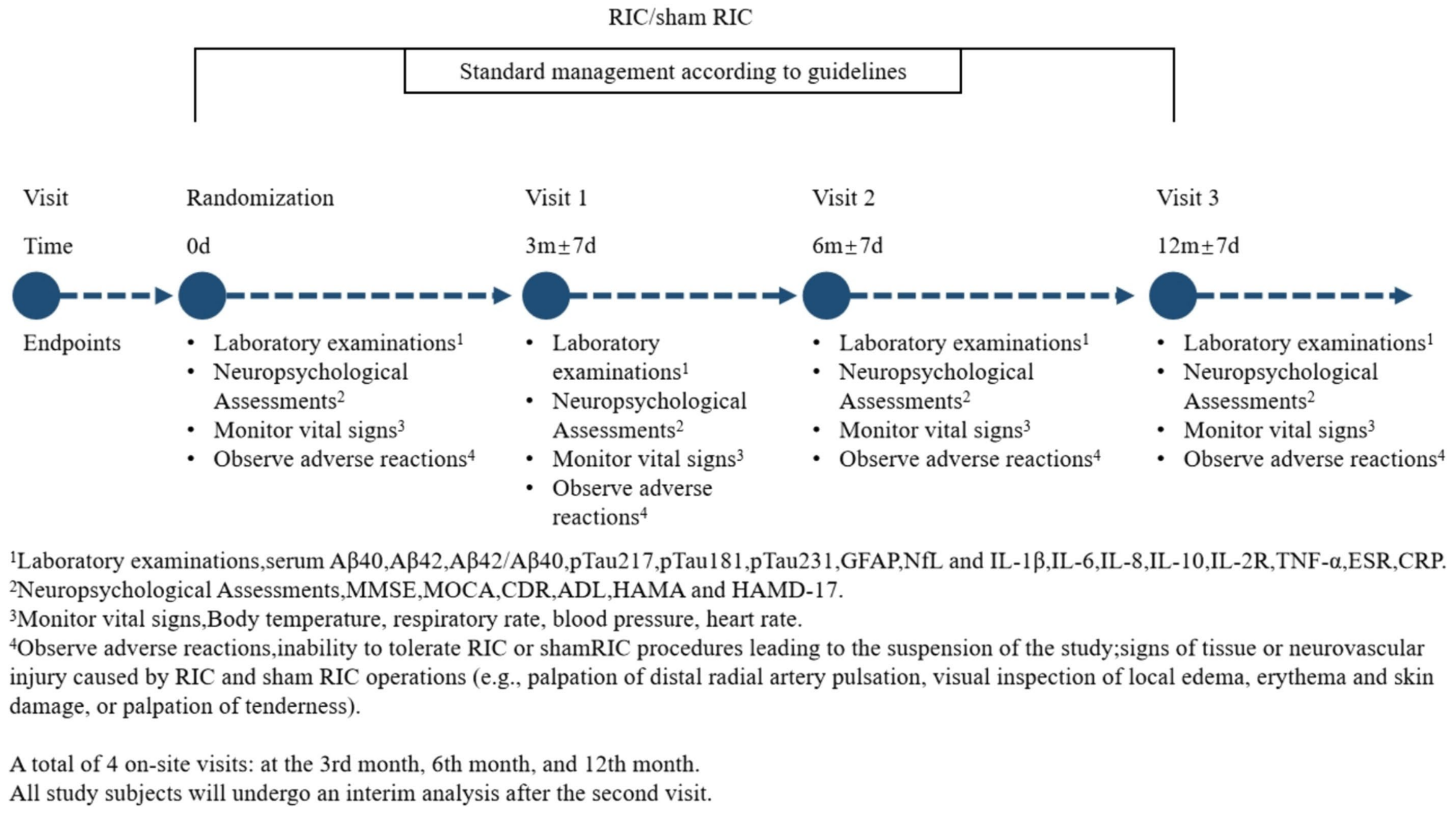
Timeline for experimental procedures.

To evaluate the long-term efficacy of RIC, the study includes a 12-month follow-up period with assessments at 6 and 12 months. These evaluations include neurocognitive scales and blood biomarkers analyses. The RIC intervention itself is administered over a 3-month period, with data collection points at baseline, 3 months, 6 months, and 12 months to systematically assess its sustained effects (Figure 2).

### Neuropsychological assessments

The Mini-Mental State Examination (MMSE), Montreal Cognitive Assessment (MOCA), and Clinical Dementia Rating (CDR) scales will be used to evaluate the cognitive function of patients with mild Alzheimer’s dementia. The Activities of Daily Living (ADL) scale will be used to assess their quality of life. The Hamilton Anxiety Rating Scale (HAMA) and the Hamilton Depression Rating Scale (HAMD-17) will be used to evaluate the mental and emotional status. These assessments will be conducted at baseline, 3 months, 6 months, and 12 months.

### Blood sample collection and analysis

Under fasting conditions, 5 mL of peripheral venous blood will be drawn, and the plasma samples will be stored in a − 80°C freezer. Enzyme-linked immunosorbent assay (ELISA) will be used to measure serum levels of interleukin-1β (IL-1β), interleukin-6 (IL-6), interleukin-8 (IL-8), interleukin-10 (IL-10), interleukin-2 receptor (IL-2R), tumor necrosis factor-*α* (TNF-α), erythrocyte sedimentation rate (ESR), C-reactive protein (CRP), and plasma biomarkers including glial fibrillary acidic protein (GFAP), phosphorylated tau at positions 181, 217, and 231 (pTau181, pTau217, and pTau231), Aβ42 and Aβ40 and their ratios, and neurofilament light chain (NfL). These measurements will be conducted at baseline, 3 months, 6 months and 12 months.

## Outcomes

### Outcomes for safety

The primary safety outcomes of this study are to evaluate the safety profile of RIC. Specifically, the following adverse events will be monitored:

1)  Psychological and Emotional Impact: Clinical observations and previous research have highlighted that patients with AD often experience feelings of helplessness and depression as their cognitive function declines and their ability to perform daily activities diminishes. These emotional challenges can lead to anxiety and fears about disease progression. Additionally, the pressure sensation caused by RIC training may exacerbate these feelings. To address this, the psychological condition of patients will be assessed using the HAMA and the HAMD-17, and the incidence of related adverse events will be calculated.
2)  Procedure Tolerability: Instances of participants being unable to tolerate either the RIC or sham RIC procedures, resulting in suspension from the study, will be recorded as adverse events.
3)  Physical Injuries: Signs of tissue or neurovascular injury potentially caused by RIC or sham RIC procedures will be monitored. This includes assessments such as palpation of the distal radial artery for pulsation, visual inspection for local edema, erythema, or skin damage, and palpation for tenderness in the affected areas.
4)  Vital Sign Monitoring: Participants’ vital signs, including body temperature, respiratory rate, blood pressure, and heart rate, will be recorded to identify any potential systemic effects of the intervention.

All adverse events will be reviewed and independently determined by trained members of the research team who are blinded to the randomization group. These outcomes aim to comprehensively evaluate the safety of RIC and ensure that any risks associated with the intervention are carefully monitored and managed.

In this study, participant safety is closely monitored through structured safety management protocols. If a participant experiences psychiatric worsening, indicated by significant increases in anxiety or depression scores (HAMA or HAMD-17), an immediate referral to a psychiatrist will be initiated, and psychiatric interventions or modifications to study procedures may be recommended as appropriate. In cases of physical injury, such as tissue damage, neurovascular compromise, or significant pain related to the cuff procedure, the intervention will be immediately paused. A multidisciplinary specialist team, including experts in neurology, cardiology, neurosurgery, and vascular surgery, will promptly evaluate the participant. Treatment will be provided as needed, and the participants will be closely monitored until full resolution. Detailed records of these events will be documented and reviewed by our Data and Safety Monitoring Board (DSMB) to ensure thorough oversight and appropriate follow-up actions.

### Outcomes for efficacy

The secondary outcomes of this study are categorized into three classes. Cognitive function and daily living abilities will be evaluated using the MMSE, MoCA, and CDR scales, while daily living abilities will be assessed using the ADL scale. Disease severity and progression will be measured through plasma biomarkers, including Aβ40, Aβ42, the Aβ42/Aβ40 ratio, pTau217, pTau181, GFAP, and NfL. Neuroinflammation levels will be assessed via serum levels of IL-1β, IL-6, IL-8, IL-10, IL-2R, TNF-*α*, ESR, and CRP. These outcomes aim to comprehensively evaluate the efficacy of RIC in addressing cognitive decline, daily living abilities, disease biomarkers, and neuroinflammation in patients with AD.

### Sample size estimation

This is a Phase I trial measuring the safety and feasibility of RIC. Since no prior clinical studies have examined RIC in AD patients, there is no reference data available. Ganesh proposed that a cohort of 12 patients per group is sufficient to assess the safety of remote ischemic conditioning in patients with Vascular Cognitive Impairment (28). Tong’s Phase I clinical trial on the safety and efficacy of RIC in stroke patients suggested that 20 cases are sufficient to assess the feasibility of a pilot study (29). Based on studies addressing the safety and feasibility of RIC, this project targets 20 patients in each group, providing sufficient confidence to proceed to a subsequent Phase II study with a randomized sham control.

### Statistical analysis

Data will be obtained from all patients who complete the study protocols and follow-ups and will be analyzed using the per-protocol (PP) approach. Statistical analysis will be performed using SPSS version 20 (SPSS Inc., Chicago, IL, United States), with a significance level of *p* < 0.05. Demographics and clinical characteristics will be analyzed using descriptive statistics. Continuous and categorical data will be reported as mean values, standard deviations, number values, and percentages. Continuous variables following a normal distribution will be compared using the independent samples *t*-test, ANOVA, or rank-sum test, while categorical variables will be compared using the chi-square test.

In our RIC trial in mild Alzheimer’s dementia (*n* = 40), we implemented two strategies appropriate for small-sample longitudinal data. First, single random forest imputation was applied to address missing cognitive scores (e.g., MoCA/MMSE) by using predictors such as baseline Aβ42/Aβ40 ratio and RIC adherence preserve variable interaction structure. Second, Generalized Estimating Equations (GEE) will be used to model outcome trajectories under the assumption of missing at random (MAR), thereby accommodating incomplete data without imputation. Attrition is minimal (7% MoCA loss at 12 months) and APOE subgroup analyses show no evidence of differential dropout. This approach balances methodological rigor with feasibility and aligns with CONSORT recommendations for early-phase trials with limited resources.

## Discussion

Ischemic-reperfusion injury has emerged as a key factor in the pathogenesis of various neurodegenerative diseases, including AD (30). This paradoxical phenomenon occurs when the restoration of blood flow to previously ischemic tissue induces cellular damage oxidative stress, and neuroinflammation, thereby exacerbating neurodegenerative processes (31). In AD, reperfusion injury is increasingly recognized as an important mechanism contributing to cognitive decline and neuronal degeneration. Recent studies have emphasized this connection. Pluta et al. highlighted the significant role of post-ischemic tau pathology in AD-related neurodegeneration, suggesting reperfusion injury may be a critical driver of disease progression (32). Ułamek-Kozioł and colleagues further supported this link by demonstrating significant proteomic and genomic alterations involving tau protein following ischemia–reperfusion events (33). Given the protective mechanisms observed with postconditioning strategies, such as reduced apoptosis, attenuated inflammatory response, and enhanced microcirculation, RIC presents a promising non-pharmacological with potential relevance to AD (34, 35). Building on these findings, the present study explores the neuroprotective efficacy of RIC in mitigating ischemia-associated cognitive decline and neurodegeneration in patients with mild Alzheimer’s dementia.

This study investigates the potential of RIC as a non-invasive, multi-target intervention for mitigating cognitive decline in mild Alzheimer’s dementia. The parameters selected for evaluation, including neuropsychological scales, plasma biomarkers, and inflammatory cytokines, provide a comprehensive framework for assessing the safety and efficacy of RIC in addressing the complex pathology of AD.

The assessment of cognitive function and daily living abilities using scales such as the MMSE, MoCA, and CDR reflects the study’s primary clinical outcomes of interest (36). These tools are widely recognized for their sensitivity and specificity in detecting changes in cognitive performance and functional status in mild Alzheimer’s dementia (8, 37), making them reliable measures for evaluating therapeutic impact of RIC. Similarly, biomarkers like Aβ40 and Aβ42, p-tau181, p-tau217, and NfL will be used to monitor disease progression and neurodegeneration (38–41). These markers are central to the pathological cascade in AD, providing insights into the molecular mechanisms modulated by RIC. Longitudinal measurement of these biomarkers enables a detailed analysis of RIC’s capacity to influence amyloid metabolism, tau pathology, and neuronal integrity (5, 42).

In addition, inflammatory cytokines, including IL-6, TNF-*α*, and CRP, will be measured to assess RIC’s effect on neuroinflammation (20, 22, 43–46). Given that neuroinflammation is a key driver of AD pathology, modulating these pathways represents a plausible mechanism through which RIC may exert its neuroprotective effects (36, 38). The inclusion of these markers facilitates a more comprehensive understanding of both systemic and central inflammatory responses to RIC.

The reliability of these parameters in supporting the study’s hypothesis is reinforced by their established roles in AD research and their responsiveness to therapeutic interventions. Neuropsychological scales are routinely employed in clinical trials to detect clinically meaningful changes in cognition and function (36). Likewise, biomarkers like Aβ and tau are well-characterized indicators of AD pathology, with fluctuations in their levels correlating with disease progression (22, 36, 38, 47, 48). Inflammatory cytokines, extensively studied as markers of immune system activity, provide insight into the inflammatory environment associated with AD (22, 48).

Furthermore, the multi-timepoint design of the study—evaluating outcomes at baseline, 3 months, 6 months, and 12 months—enhances the robustness of the findings. This approach allows for the assessment of both immediate and sustained effects of RIC, ensuring that observed changes result from the intervention rather than transient or external factors.

## Implications of findings

By integrating cognitive, functional, and biomarker assessments, this study establishes a rigorous framework for evaluating the therapeutic potential of RIC in mild Alzheimer’s dementia. The consistent use of validated scales and biomarkers ensures that the findings are both clinically relevant and scientifically credible. If successful, this research could provide a foundation for future studies exploring the broader applicability of RIC in neurodegenerative conditions.

This study has several limitations. First, the RIC treatment protocol used is pragmatic and tailored to individuals with mild dementia due to Alzheimer’s. Second, the study population is limited to patients in the mild stage of Alzheimer’s dementia meeting NIA-AA diagnostic criteria, which may introduce selection bias by excluding moderate-to-severe cases. This cohort restriction could underestimate both therapeutic efficacy (e.g., disease-modifying effects) and adverse events (e.g., cerebrovascular complications), potentially affecting the risk–benefit assessment in broader AD populations. Third, the absence of longitudinal data limits generalizability to disease progression patterns observed in advanced stages. Despite these limitations, our findings are consistent with emerging evidence supporting the neuroinflammatory modulation potential of RIC, emphasizing the need for multicenter trials with extended follow-up to validate and expand upon these preliminary results.

## Conclusion

This study’s design is integral not only to assessing RIC’s safety and efficacy but also establishes reliable indicators of its potential to modulate key pathophysiological mechanisms underlying AD progression. The strategic selection of outcome measures underscores the study’s scientific rigor and its capacity to support the hypothesis that RIC can mitigate cognitive decline in mild Alzheimer’s dementia.

## Glossary

RIC: Remote Ischemic Conditioning
AD: Alzheimer’s disease
HAMA: Hamilton Anxiety Rating Scale
HAMD-17: Hamilton Depression Rating Scale
MMSE: Mini-Mental State Examination
MoCA: Montreal Cognitive Assessment
CDR: Clinical Dementia Rating
ADL: Activities of Daily Living
Aβ: β-amyloid
VCI: Vascular cognitive impairment
DSMB: Data and Safety Monitoring Board
NIA-AA: National Institute on Aging and Alzheimer’s Association
ELISA: Enzyme-linked immunosorbent assay
IL-1β: Interleukin-1β
IL-6: Interleukin-6
IL-8: Interleukin-8
IL-10: Interleukin-10
IL-2R: Interleukin-2 receptor
TNF-α: Tumor necrosis factor-α
ESR: Erythrocyte sedimentation rate
CRP: C-reactive protein
GFAP: Glial fibrillary acidic protein
NfL: Neurofilament light chain
PP: Per-protocol
MAR: Missing at random
